# Prescriptions of antidepressants and anxiolytics in survivors of out-of-hospital cardiac arrest - a nationwide register-based follow-up study

**DOI:** 10.1186/1757-7241-23-S1-A10

**Published:** 2015-07-16

**Authors:** Kristian Bundgaard, Kristian Kragholm, Rikke N Mortensen, Steen M Hansen, Christian Torp-Pedersen, Bodil S Rasmussen

**Affiliations:** 1 Department of Anesthesiology and Intensive Care Medicine, Aalborg University Hospital, Aalborg, Denmark; 2 Department of Health, Science and Technology, Aalborg University, Aalborg, Denmark; 3 Department of Clinical Epidemiology, Aalborg University Hospital, Aalborg, Denmark

## Background

The need for treatment with antidepressants and anxiolytics in survivors of out-of-hospital cardiac arrest (OHCA) is not known. Such use is an important part of evaluating neurological outcome in OHCA survivors and we have therefore performed a nationwide study.

## Methods

Nationwide data on OHCA from the years 2001-2011 was available from the Danish Cardiac Arrest Registry. We linked data to the Danish National Prescription Registry and excluded patients with prior prescriptions of antidepressants and/or anxiolytics within half a year before OHCA. We calculated time to the first prescription of antidepressants and/or anxiolytics following OHCA. Multivariable analyses were carried out using cause-specific Cox regression methods, taking competing risk of death into consideration.

## Results

From 2,469 30-day OHCA survivors, we included 2,018 cases (median age 62 (IQR 53-71), 79.7% men) in the study population, of which 242 (11.2%) were prescribed an antidepressant and 165 (8.2%) were prescribed an anxiolytic drug during the first year after OHCA. Stratified by years (2001-2005 vs. 2006-2011), 77 (12.5%) versus 165 survivors (11.8%) were prescribed an antidepressant (p = 0.92), and 62 (8.2%) versus 103 (7.4%) survivors were prescribed an anxiolytic drug (p = 0.78). Cardiac arrest witnessed by bystander (HR 0.63 (95% CI 0.43-0.93, p = 0.019) and bystander cardiopulmonary resuscitation (HR 0.73 (95% CI 0.53-0.99, p = 0.045), were in multivariable analyses associated with less prescriptions of antidepressants within follow-up period. Similar results were seen in prescriptions of anxiolytics: cardiac arrest witnessed by bystander (HR 0.57 (95% CI 0.36-0.90, p = 0.015), and bystander cardiopulmonary resuscitation (HR 0.57 (95% CI 0.39-0.83, p = 0.003). Age above 74 years, Charlson comorbidity index score above 0 and non-shockable initial heart rhythm were associated with death during the follow-up period.

## Conclusions

Overall, 11.2% of the survivors were prescribed an antidepressant drug and 8.2% were prescribed an anxiolytic drug during the first year after OHCA. Cardiac arrests witnessed by bystander and bystander cardiopulmonary resuscitation were in our multivariable models associated with less prescriptions of antidepressants and anxiolytics, stressing the importance of early, prompt interventions in OHCA care on outcome.

